# Efficacy of a Mobile-Enabled Web App (iCanFit) in Promoting Physical Activity Among Older Cancer Survivors: A Pilot Study

**DOI:** 10.2196/cancer.4389

**Published:** 2015-06-26

**Authors:** Yan Alicia Hong, Daniel Goldberg, Marcia G Ory, Samuel D Towne Jr, Samuel N Forjuoh, Debra Kellstedt, Suojin Wang

**Affiliations:** ^1^ School of Public Health Texas A&M University College Station, TX United States; ^2^ Department of Geography Department of Computer Science and Engineering Texas A&M University College Station, TX United States; ^3^ Department of Family Medicine Baylor Scott & White Health Temple, TX United States; ^4^ Department of Statistics Texas A&M University College Station, TX United States

**Keywords:** physical activity, mobile health, older adults, cancer survivors, iCanFit, pilot

## Abstract

**Background:**

The benefits of physical activity for cancer survivors are well documented. However, few older cancer survivors are engaged in regular physical activity. Mobile technologies may be an effective method to deliver physical activity promotion programs for older cancer survivors. *iCanFit,* a mobile-enabled Web-based app, was developed based on formative research and usability testing. This app includes interactive features of physical activity, goal setting and tracking, and receiving personalized visual feedback.

**Objective:**

The aim of this study is to pilot test the initial efficacy of iCanFit.

**Methods:**

Older cancer survivors (N=30) were recruited online through our collaborative partnership with a cancer survivor's organization. After the participants completed an online baseline survey, they were asked to use the iCanFit website. Instructional videos on how to use the web app were available on the website. Participants were asked to complete a follow-up survey 2-3 months later. Participants’ physical activity, quality of life, and their experience with iCanFit were measured.

**Results:**

A total of 30 participants completed the baseline survey, and 26 of them (87%, 26/30) also completed a follow-up survey 2-3 months later. The median age of participants was 69 years (range 60-78). Participants’ quality of life and engagement in regular physical activity improved significantly after the use of iCanFit. Participants indicated a general affinity towards the key function “Goals” in iCanFit, which motivated continued activity. They also provided suggestions to further improve the app (eg, adding a reminder functionality, easier or alternative ways of entering activities).

**Conclusion:**

The interactive Web-based app iCanFit has demonstrated initial efficacy. Even though our study was limited by a small sample size, convenience sampling, and a short follow-up period, results suggest that using mobile tools to promote physical activity and healthy living among older cancer survivors holds promise. Next steps include refining iCanFit based on users’ feedback and developing versatile functionality to allow easier physical activity goal setting and tracking. We also call for more studies on developing and evaluating mobile and web apps for older cancer survivors.

## Introduction

More than 87% of American adults have used the Internet. Even among older adults, 88% of those aged 50-64 are online and more than 57% of those older than 64 are online. Furthermore, mobile phone use among older adults has also grown from 11% in 2011 to 18% in 2013 [1]. Literature suggests that older adults are increasingly turning to the Internet for information [2]. Despite high rates of Internet access and mobile device ownership, only a small number of mobile or Web-based apps have been designed specifically for older adults.

Physical activity is particularly important for older adults, especially for those with chronic conditions including cancer [3]. Regular physical activity has been shown to effectively lessen fatigue and improve overall quality of life for cancer survivors [4]. Web- or mobile-based physical activity interventions have been shown to be effective [5-8], but such interventions have rarely targeted older adults, and web or mobile apps designed specifically for older cancer survivors are especially scarce [9,10].

Early detection and improved treatment for cancer have resulted in approximately 13 million survivors being alive in the United States today; this number will increase by nearly a third to almost 18 million by 2022 [11]. A majority of cancer survivors are >60 years of age. Caring for the large number of older cancer survivors presents a significant public health challenge [12]. The benefits of physical activity for cancer survivors have been reinforced through several guidelines [12,13]. However, the level of adherence to physical activity guidelines among cancer survivors is very low. For example, only 4.6% of breast cancer survivors followed physical activity guidelines compared to 12% of women without breast cancer [14]. The level of physical activity among older cancer survivors is believed to be even lower [13]. Thus, there is an urgent need for effective interventions that can reach large numbers of older cancer survivors efficiently. Literature suggests that older cancer survivors are more likely to use the Internet compared to their counterparts without a cancer diagnosis [15]. Once online, cancer survivors are more likely to use the Internet for health-related purposes [16]. Thus, it is potentially efficient and effective to deliver Web- or mobile-based interventions to older cancer survivors.

Prior studies on promoting physical activity for older adults suggested that goal setting is an effective intervention strategy. Through setting specific and achievable goals and regular feedback, people are motivated to exercise. Additionally, personalized feedback reinforces maintenance of behavioral change [17,18].

**Figure 1:**
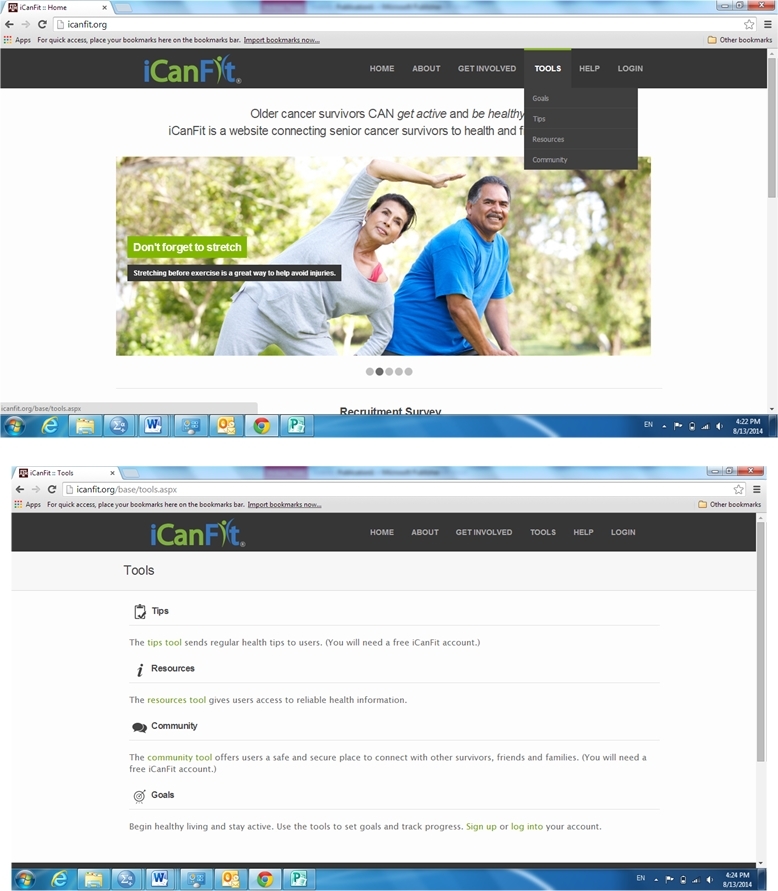
Screenshot of iCanFit.

**Figure 2:**
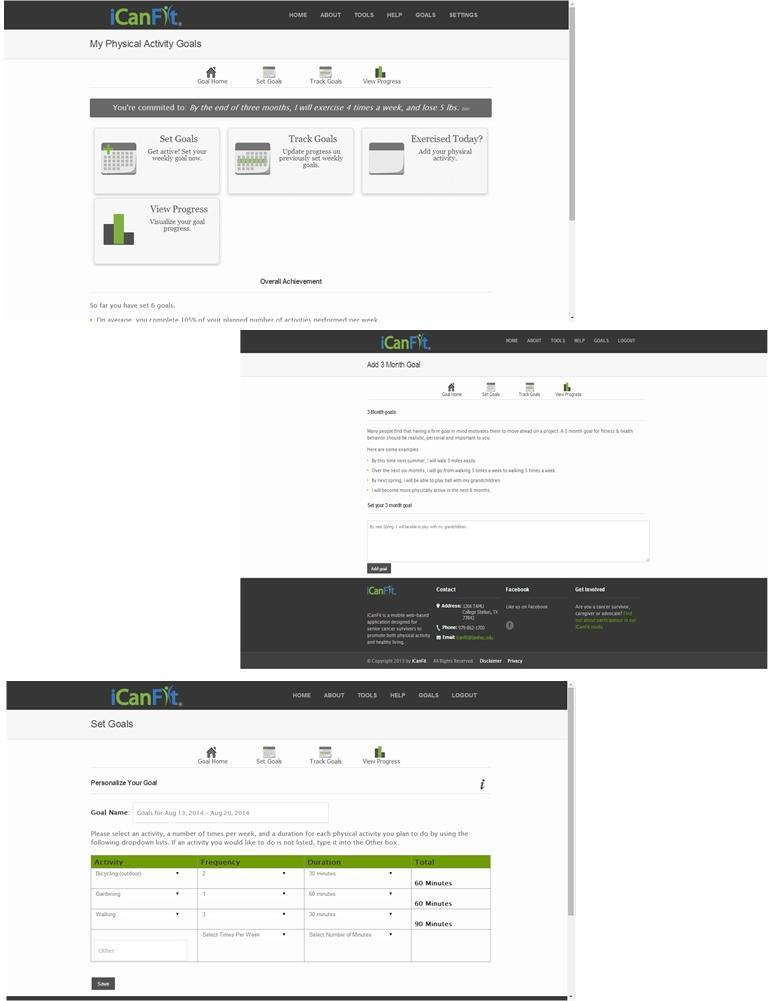
"Goals" function of iCanFit.

**Figure 3:**
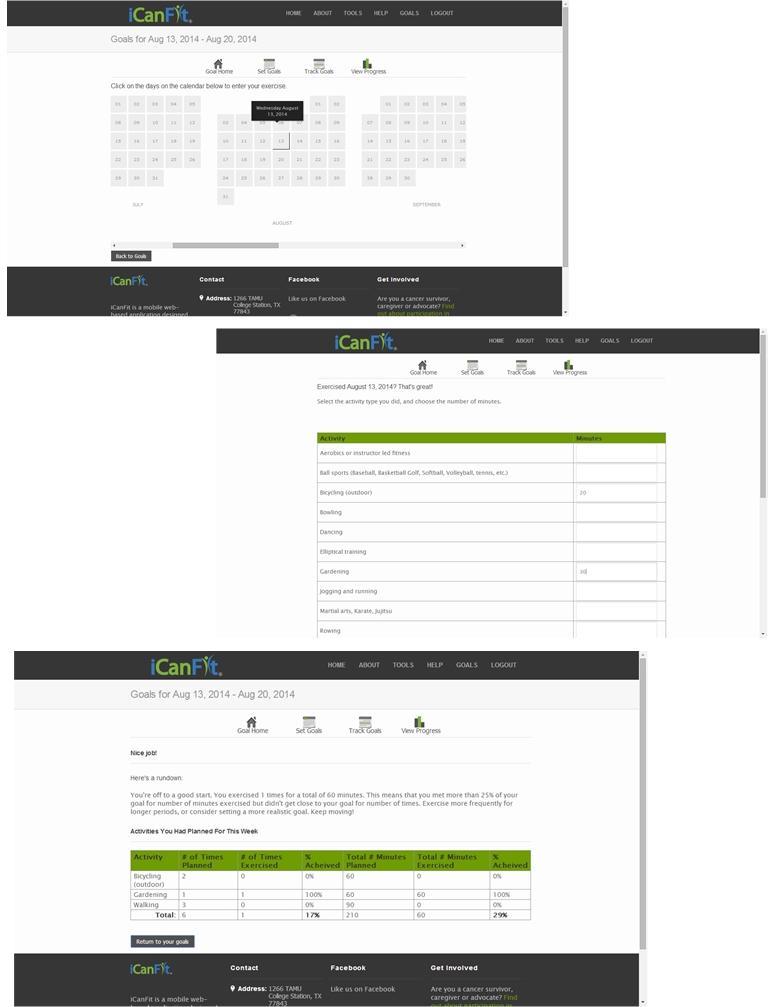
Activity log of iCanFit.

With the aim of promoting physical activity among older cancer survivors, the mobile-enabled website app iCanFit was designed with formative research with key stakeholders [19], along with usability and acceptability testing [20]. The major functions in the iCanFit web app include “Goals” (physical activity, goal setting, and tracking), “Community” (an online network for users), “Tips” (regularly updated tips on healthy living), and “Resources” (active links to reliable health information) (Figure 1). Of these functions, “Goals” is the most important tool (Figure 2). Guided by the Theory of Goal Setting [18], “Goals” motivates participants to exercise regularly through goal setting, activity tracking, personalized feedback, and progress reviews. A new participant will be cued to set up a long-term goal; then be cued to set a short-term, usually weekly, goal. Examples of goals are available to guide participants set specific and tangible goals. Each goal includes type, frequency, and duration of the physical activities. The system will automatically calculate total minutes and energy expenditure for each and all of the activities (Figure 2). On an interactive calendar, participants can enter their activity and log the total number of minutes they exercised on a selected day (Figure 3). Their activity log will be compared to their goals and they will receive tailored messages based on this comparison. Examples of such messages are “Congratulations, you’ve achieved your goal, keep up the good work!” or

Sorry you did not meet your goal. You may consider setting a more realistic goal. Keep moving!

**Figure 4:**
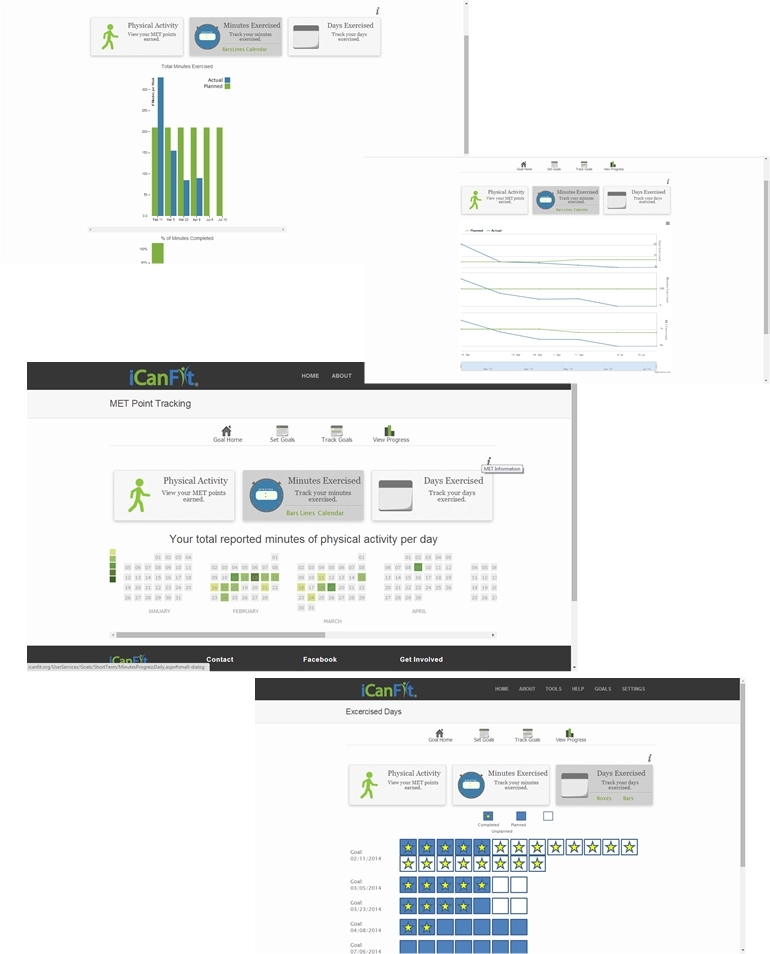
"View Progress" function of iCanFit.

The tailored messages (Figure 3) are sent automatically from iCanFit using a pre-designed database that contains >100 messages for different conditions of meeting goals. Finally, “View Progress” allows users to track their progress through various metrics, including total energy expenditure, total minutes exercised, number of days exercised, and comparisons between actual activity and their preset goals (Figure 4). Users have the options to view their progress as bars, lines, and/or as a calendar (Figure 4).

This study targeted the understudied but growing population of older cancer survivors, and explored the feasibility and initial efficacy of an interactive Web-based app to promote their physical activity. Through a pilot test of pre-post design, we aimed to answer the research question of whether participating in iCanFit is associated with changes in physical activity and quality of life among older cancer survivors.

## Methods

### Overview of Study Design

This was a pre-post design study with 2-3 months of follow-up. Online surveys and a mobile-enabled web app were used to collect participant data.

### Participant Recruitment

All participants were recruited online. Through our collaborative partnership with a cancer survivor's organization, emails were sent out to their list-serve inviting eligible cancer survivors to participate. Inclusion criteria for participation included (1) ≥60 years, (2) having ever been diagnosed with cancer, (3) reporting the ability to do physical activity, and (4) having access to the Internet. Potential participants were instructed to the iCanFit website [21]. On the homepage, there was a screening survey for potential participants. Individuals who completed the screening survey (including the provision of contact information) received a phone call from our research staff to re-verify their eligibility. Eligible participants received instruction on how to participate in the study. Instructional videos on how to use iCanfit were also available on the “Help” page.

### Data Collection Procedures

Project information was sent out to participants via email with a link to an online survey. Prior to the survey, an informed consent with a detailed project description, benefits, and potential risks of participation was provided. Participants who completed the informed consent were directed to the baseline survey, which took approximately 10 minutes to complete. After the survey, participants were directed to the project site and instructed to create a username and password. The “Help” page also contained instructional videos on how to use the web app. All participants were instructed to use iCanFit for about 8-12 weeks. During that time, 4-6 emails were sent to participants reminding them to continue using iCanFit. The emails were sent automatically and contained personalized information such as participant’s name, duration of the study, and when the follow-up survey would be sent. After 8-12 weeks, participants were sent a link to the follow-up survey, which took approximately 10 minutes to complete. Participants who completed the baseline survey received a $15 gift card and those who completed the follow-up survey received an additional $35 gift card. The study protocol was approved by the Institutional Review Board (IRB) of the Texas A&M University.

### Measures

The baseline survey included participant demographics, use of mobile tools, quality of life, and current physical activity. The quality of life was measured with 7 items, including self-rated health (1 being poor and 5 being excellent), overall quality of life, fatigue, pain, shortness of breath, stress, and sleep. The response options ranged from 0-10, with 0 being the worst and 10 the best. The Cronbach alpha value for the 7 quality of life items was .79. We also asked the participants how many days in the past 30 days did they not have good physical health, good mental health, or could not do usual activities.

Participants were asked about their current engagement in regular physical activity with the following 5 response options (1) not engaged in physical activity and have no plan of doing so, (2) not engaged but plan to do so in 3 months or less, (3) engaged occasionally but not on a regular basis, (4) engaged in regular physical activity but started less than 3 months, and (5) engaged in regular physical activity and has been doing so for more than 3 months.

In the follow-up survey, participants were asked about the same outcomes on quality of life and engagement in regular physical activity, as well as their experience with the program. Some open-ended questions were included to solicit their feedback and suggestions for iCanFit. In addition to the above self-reported subjective data, users’ activities on iCanFit including the number of goals set and achieved were captured by the web app.

### Data Analysis

All data from the online surveys were downloaded and stored in SPSS 22.0. Descriptive statistics were used for data analysis; paired *t* test was used to compare pre-post changes and assess level of significance at the *P*<.05 level. Open-ended responses were entered into Microsoft Word to identify ranges and patterns of responses.

## Results

### Demographics and Mobile Technology Use

Of the total eligible participants who created an online account and completed the baseline survey (N=30), 87% (26/30) completed the follow-up survey. These 26 participants were the sample to test the efficacy of iCanFit.

As shown in Multimedia Appendix 1, the median age of the 26 participants in the baseline sample was 69 years (range 60-78). About 70% (18/26) of them were female, 73% (19/26) were white, 77% were married (20/26), 38% (10/26) had some college education, and 42% (11/26) had completed college. Only 2 participants (7%, 2/26) were still in cancer treatment, while the remaining majority weremajority was not, including 46% (12/26) that were being monitored but not in treatment, and 46% (12/26) that were post-treatment survivors. All participants had other chronic conditions, including high blood pressure (46%, 12/26), heart disease (23%, 2/26), arthritis (31%, 8/26), osteoporosis (23%, 6/26), sciatica (12%, 3/26), diabetes (12%, 3/26), chronic back pain (12%, 3/26), and depression or anxiety (8%, 2/26). About 46% (12/26) of participants talked to their health care providers about physical activity almost every time they saw the provider, 27% (7/26) said sometimes they did, and 23% (6/26) said they rarely or never did.

All of the participants had high-speed Internet access at home, and most of them used multiple mobile tools for Internet access. Specifically, 69% (18/26) used a desktop, 46% (12/26) used a laptop, 46% (12/26) used a tablet, and 81% (21/26) used a mobile phone with app capabilities; and as many as 23% (6/23) of participants used a mobile phone for Internet access every day. Their weekly online hours varied from 1-60 hours (median 10 hours). Most older cancer survivors frequently searched health information online, for example, only 1 person (4%, 1/26) never searched health information online, 8% (3/26) did so twice a month, 19% (2/26) did it once a week, 27% (5/26) did it 2-3 times a week, and 15% (6/26) did it every day. Most participants were active users of social media. For instance, 65% (17/26) of them were on Facebook, 50% (13/26) subscribed to an email listserv, 31% (15/26) used LinkedIn, and 19% (5/26) used an online messenger.

### Physical Activity and Quality of Life

The differences in quality of life and physical activity at baseline and follow-up are shown in Table 1. The mean self-rated health score (1-5) increased from 3.23 to 3.81, the overall quality of life score increased from 8.12 to 8.50, fatigue-related quality of life score increased from 6.04 to 6.77, pain-related quality of life score increased from 8.85 to 9.11, shortness of breath-related quality of life score increased from 8.65 to 9.04, stress-related quality of life score increased from 6.27 to 7.04, and sleep quality also increased from 6.19 to 7.00. All of the quality of life-related changes were significant (*P*<.05). The number of days without good physical health, good mental health, or reporting they could not do usual activities did not have significant changes due to flooring effects of the numbers.

**Table 1:** Efficacy of iCanFit on promoting physical activity and quality of life among older cancer survivors.

Outcome measure		Baseline (N=26)	Follow-up (N=26)	*P* value
**Quality of life, mean score**				
	Self-rated health (1-5)	3.23	3.81	.0001
	Overall quality of life (0-10)	8.12	8.5	.004
	Fatigue (0-10)	6.04	6.77	.002
	Pain (0-10)	8.85	9.11	.016
	Shortness of breath (0-10)	8.65	9.04	.023
	Stress (0-10)	6.27	7.04	.0013
	Sleep (0-10)	6.19	7.0	.013
	Number of days without good physical health in past 30 days	3.57	1.65	.06
	Number of days without good mental health in past 30 days	1.54	1.19	.07
	Number of days could not do usual activities in past 30 days	1.54	0.88	.07
**Current level of physical activity, n (%)**				
	Not engaged in physical activity and have no plan	3 (12%)	0	.083
	Not engaged in physical activity but plan to do so in 3 months	5 (19%)	0	.022
	Engaged in physical activity occasionally, but not on a regular basis	7 (27%)	5 (19%)	.77
	Engaged in regular physical activity, but started less than 3 months	0	5 (19%)	.022
	Engaged in regular physical activity, and has been doing so for 3 months	11 (42%)	15 (58%)	.043

In terms of self-reported physical activities at baseline, 3 participants (12%, 3/26) were not engaged in physical activity and had no plan of doing so and 5 participants (19%, 5/26) were not engaged in physical activity but planned to do so in less than 3 months. All of these 8 participants were engaged in physical activity in the follow-up. The number of participants engaged in regular physical activity also increased from 11 (42%, 11/26) to 15 (58%, 15/26).

### Experience With iCanFit

Participants’ use of and experience with iCanFit is depicted in Table 2. Most participants accessed iCanFit with multiple mobile devices including 58% (15/26) via desktop, 38% (10/26) via laptop, 23% (6/26) via tablet, and 27% (7/26) via mobile phones. Participants used iCanFit with varying frequencies; 12% (3/26) used it once every 2 weeks, 62% (16/26) used it once a week, 19% (5/26) used it 2-3 times per week, and 8% (2/26) used it 4-5 times a week. Their weekly median time on iCanFit was 25 minutes (range 5-60). About 65% (17/26) of participants had talked to their family and friends about iCanFit, and 27% (7/26) used iCanFit with their family or friends.

**Table 2:** User experience with iCanFit (follow-up, N=26).

Measure		n (%)
**Mode of accessing iCanFit**		
	Desktop	15 (58%)
	Laptop	10 (38%)
	Mobile phone	7 (27%)
	Tablet	6 (23%)
**How often did you log into iCanFit?**		
	Once every 2 weeks	3 (12%)
	Once or twice per week	16 (62%)
	2-3 times per week	5 (19%)
	4-5 times per week	2 (8%)
	Every day	0
Minutes on iCanFit each week, median (range)		25 (5-60)
Talked to family or friends about iCanFit, n (%)		17 (65%)
Used iCanFit with family or friends, n (%)		7 (26.9%)
**Ease of use iCanFit (1-5), mean (SD)**		
	Overall	4.1 (1.7)
	Goals	3.9 (2.1)
	Healthy tips	4.2 (0.9)
	Resources	4.5 (0.8)
	Community	4.1 (0.9)

Table 2 also demonstrates that participants reported an overall positive experience with iCanFit. On the scale of 0-5, the mean (SD) value of overall ease of using iCanFit was 4.1 (1.7), 3.9 (2.1) for "Goals", 4.2 (0.9) for "Healthy Tips", 4.5 (0.8) for "Resources", and 4.1 (0.9) for "Community".

Participants also shared their experience of using iCanFit. Most participants made comments like “Great program, it helps me track my exercise.” Their favorite functions included the graphic display of their progress and the personalized feedback of their physical activities. For example, one shared “I like it sends me feedback right after I enter my activities,” and “It’s great to see a bar chart comparing my goals and my activities.” Participants who did not exercise regularly or have an exercise plan prior to the intervention reported using iCanFit to set goals and track their activities. For example, one participant reported, “It motivates me to exercise more so I can meet or exceed my goals,” and “After some weeks of using it, I know how much I can do on a weekly basis.”

Participants also shared suggestions on further improving the programs. Nearly half of the participants had frustration with forgetting to enter activities. “I often forget to enter my activities, and at the end of the week, I would totally forget what I’ve done,” and “It’d be better if I don’t need to enter my activity because I easily forget.” Some suggested using automatic function; for example, a participant had proposed a pedometer “If you could link my pedometer to iCanFit, then I don’t need to enter activity.” Participants also commented that they mostly used the "Goals" function and didn’t use the other functions (Healthy tips, Resources and Community) very often.

Within2-3 months of using iCanFit, participants set a total of 289 goals, with a mean of 11.1 per participant. Nearly half of these goals were met. The most typical activities included walking, jogging, aerobics, and gardening.

## Discussion

### Principal Findings

To our knowledge, there has been little systematic research of mobile-enabled, Web-based apps for older cancer survivors. As one of the first pilot studies, the initial efficacy of iCanFit as a web app has been demonstrated in this study. Significant improvement in quality of life and engagement in physical activity all indicate that this, and similar mobile-enabled, web apps may, be able to have positive effects for older cancer survivors. Positive user experiences with iCanFit, coupled with improvements in health-related items all point to the promising utility of this interactive web app. The already large, parallel advances in similar technology including mobile and web apps, fitness trackers, and other devices, may potentially benefit millions of older adults with chronic conditions.

### Limitations

Our study has the following limitations. First, the use of convenience sampling, the sample being mostly drawn from a cancer survivor's organization in Texas, and the relatively highly educated sample (eg, most with some college education or above) limits generalizability to all older cancer survivors in the country. In addition, there was a voluntary bias in the sampling as the older cancer survivors who signed up for this study might have been those who were already physically active. Second, our measures of quality of life were mainly developed from our own research and might not be comparable to other studies with validated quality of life scales. The pilot study described here aimed to test the initial efficacy of iCanFit and it took participants <10 minutes to complete the online survey. Third, the quality of life measures focused on physical and mental health functioning and not a more broad application of multiple quality of life domains. Even so, lifestyle factors such as physical activity were the focus of this study. Future studies need to include validated, multi-dimensional qualify of life scales to increase its comparability to other studies. Fourth, the measure of physical activity was dependent on self-entry and self-report, which may incur recall or social desirability biases. Future research should thus include alternative forms of data entry. For example, the recent popularity of accelerometer and sensors can be used to capture users’ physical activity to reduce users’ burden and potential errors and biases in data entry. Finally, our study did not have a control group and had only one follow-up. Future studies need to include a larger sample, a control group, and long-term follow-ups to further establish the efficacy of the intervention. While the vast majority of older adults have at least one chronic condition, nearly three quarters have ≥2 [22]. Thus, effective interventions must be multi-faceted and recognize that older adults are likely to have co-morbidities which bring greater complexity to adopting and maintaining healthy lifestyles [23,24]. In this study, all older cancer survivors had multiple chronic conditions, which is important to consider when determining their specific needs and barriers to physical activity [25,26]. For example, more tailored messages about how to manage chronic conditions and tips on easy access to opportunities for physical activity and enhanced communication with healthcare providers may be helpful [27-29]. We also need to explore innovative multicomponent interventions to tailor the needs of older adults [30]. Online and mobile health programs designed for older adults need to tailor for their literacy level, cognitive function, and physical abilities [31].

### Conclusions

Since the population of older cancer survivors is expected to grow; interventions to improve quality of life among this population are timely and will continue to be needed at even greater levels. The high rates of Internet access and mobile phone ownership throughout the United States and globally have driven greater searches for health-related information online via various mobile devices. Thus, the utility of this timely research is of global interest. Our next steps will be focused on two aspects: building the mobile app with support for communication with smart devices (eg, devices with sensors), and disseminating this mobile-enabled app to greater numbers of older cancer survivors.

## Appendix

Multimedia Appendix 1 Demographics, health conditions, and use of mobile tools among participants (baseline and follow-up).
