# Supplementary material for: Efficacy of a Mobile-Enabled Web App (iCanFit) in Promoting Physical Activity Among Older Cancer Survivors: A Pilot Study
Source: JMIR Cancer. 2015 Jun 26;1(1):e7. doi: 10.2196/cancer.4389 (PMC5367673; doi:10.2196/cancer.4389)
Supplement: Multimedia Appendix 1 [file cancer_v1i1e7_app1.pdf]

Demographics	N (%)
Age, median (range)	69 (60~78)
Gender	
Male	8 (30.7%)
Female	18 (69.2%)
Race/Ethnicity	
White	19 (73.0%)
Hispanic	2 (7.6%)
African American	5 (19.2%)
Others	0
Marital status	
Married	20 (76.9%)
Unmarried	6 (23.0%)
Education	
College or above	11 (42.3%)
Some college	10 (38.4%)
Less than college	5 (19.2%)
High speed internet access at home	26 (100%)
Years of cancer survivorship, median (range)	9.5 (0.5~50)
Cancer treatment status	
In treatment	2 (7.6%)
Being monitored but not in treatment	12 (46.1%)
Post-treatment survivor	12 (46.1%)
Other chronic conditions	
High blood pressure	12 (46.1%)
Heart disease	6 (23.0%)
Arthritis	8 (30.7%)
Osteoporosis	6 (23.0%)
Sciatica	3 (11.5%)
Diabetes	3 (11.5%)
Chronic back pain	3 (11.5%)
Depression or anxiety	2 (7.6%)
Other chronic conditions	7 (26.9%)
Mobile tools for internet access	
Desktop	18 (69.2%)
Laptop	12 (46.1%)
Tablet	12 (46.1%)
Smart phone	21 (80.7%)
Hours online per week, median (range)	20 (1~60)
Frequency of searching health information online	
Never	1 (3.8%)
Less than once a month	2 (7.6%)
Twice a month	5 (19.2%)
Once a week	7 (26.9%)
Two to three times a week	7 (26.9%)

Everyday	4 (15.3%)
Use of social media	
Facebook	17 (65.3%)
Twitter	2 (7.6%)
Linkedin	8 (30.7%)
Google+/Yahoo Chat/MSN, instant messenger	5 (19.2%)
Blogs	1 (3.8%)
List-serve or email	13 (50.0%)
Frequency of using smart phone for Internet access	
Never	6 (23.0%)
Less than once a month	4 (15.3%)
About twice a month	3 (11.5%)
About once a week	2 (7.6%)
Two or three times a week	5 (19.2%)
Everyday	6 (23.0%)
Communication with doctor about physical activity	
Almost every time I see him/her	12 (46.1%)
Often	1 (3.8%)
Sometimes	7 (26.9%)
Once or twice	4 (15.3%)
Never	2 (7.6%)
